# Transcriptome analysis and cytochrome P450 monooxygenase reveal the molecular mechanism of Bisphenol A degradation by *Pseudomonas putida* strain YC-AE1

**DOI:** 10.1186/s12866-022-02689-6

**Published:** 2022-12-09

**Authors:** Adel Eltoukhy, Yang Jia, Imane Lamraoui, M. A. Abo-Kadoum, Omar Mohammad Atta, Ruth Nahurira, Junhuan Wang, Yanchun Yan

**Affiliations:** 1 Botany and Microbiology Department, Faculty of Science, Al-Azhar University, Assiut, 71524 Egypt; 2 Graduate School of Chinese Academy of Agricultural Sciences, Beijing, 100081 China; 3 National and Local Joint Engineering Research Center of Ecological Treatment Technology for Urban Water Pollution, and Zhejiang Provincial Key Lab for Subtropical Water Environment and Marine Biological Resources Protection, Wenzhou University, Wenzhou, 325035 China; 4 Higher National School of Biotechnology “Toufik Khaznadar” (ENSB), 25000 Constantine, Algeria; 5 Faculty of Science, Kabale University, Kabale, Uganda

**Keywords:** Bisphenol A, *Pseudomonas putida* YC-AE1, Cytochrome P450, Degradation pathway, RNA sequencing

## Abstract

**Background:**

Bisphenol A (BPA) is a rapid spreading organic pollutant that widely used in many industries especially as a plasticizer in polycarbonate plastic and epoxy resins. BPA reported as a prominent endocrine disruptor compound that possesses estrogenic activity and fulminant toxicity. *Pseudomonas putida* YC-AE1 was isolated in our previous study and exerted a strong degradation capacity toward BPA at high concentrations; however, the molecular degradation mechanism is still enigmatic.

**Results:**

We employed RNA sequencing to analyze the differentially expressed genes (DEGs) in the YC-AE1 strain upon BPA induction. Out of 1229 differentially expressed genes, 725 genes were positively regulated, and 504 genes were down-regulated. The pathways of microbial metabolism in diverse environments were significantly enriched among DEGs based on KEGG enrichment analysis. qRT-PCR confirm the involvement of BPA degradation relevant genes in accordance with RNA Seq data. The degradation pathway of BPA in YC-AE1 was proposed with specific enzymes and encoded genes. The role of cytochrome P450 (CYP450) in BPA degradation was further verified. Sever decrease in BPA degradation was recorded by YC-AE1 in the presence of CYP450 inhibitor. Subsequently, CYP450*bisdB* deficient YC-AE1 strain △ *bisdB* lost its ability toward BPA transformation comparing with the wild type. Furthermore, Transformation of *E. coli* with pET-32a-*bisdAB* empowers it to degrade 66 mg l^−1^ of BPA after 24 h. Altogether, the results showed the role of CYP450 in biodegradation of BPA by YC-AE1.

**Conclusion:**

In this study we propose the molecular basis and the potential role of YC-AE1cytochrome P450 monooxygenase in BPA catabolism.

**Supplementary Information:**

The online version contains supplementary material available at 10.1186/s12866-022-02689-6.

## Introduction

Bisphenol A (BPA), discovered in 1891 [1], is a ubiquitous substance widely used in many industrial applications, especially as a plasticizer in polycarbonate plastic and epoxy resins [2, 3]. Thus, in the last decade, the global production of BPA has progressively increased to meet the world demand, with approximately 6.5 million tons in 2012 [4, 5].

BPA has been regarded as one of the endocrine disruptor chemicals with estrogenic activity [6]. Numerous studies have shown reproductive toxicity and mortality in aquatic animals as a result of BPA exposure [7, 8]. Likewise, BPA, with other organic pollutants in sewage treatment plants, exerts severe damage to the cells and reproductive system in rainbow trout [9]. BPA has been evaluated in hazardous waste landfill leachates [10], industrial wastewater [11], and even drinking water [12]. Due to rapid spreading of BPA in aquatic environments and its ability to reach far places, great concern has been given to the toxicity of BPA in such environments. Several strategies have been adopted to dampen the organic pollutants burden, including oxidation [13], biodegradation [14], photoelectrocatalytic oxidation [15, 16], and adsorption [17, 18]. Interestingly, biodegradation is an eco-friendly approach with economic efficiency [14, 19].

in previous studies, bacteria with BPA-degrading capacity such as *Sphingobium* sp. YC-JY1 [20], *Pseudomonas putida* strain YC-AE1 [21], *Bacillus megaterium* strain ISO-2 [22], *Pseudomonas* sp and *Sphingomonas* sp [4], *Arthrobacter* sp. strain YC-RL1 [23] and *Achromobacter xylosoxidans* strain B-16 [24] have been reported. Most of these studies focused only on the organism used for the biodegradation of BPA and maximizing the degradation capacity of the organism. However, the underlying molecular mechanisms of the degradation process remain unclear. It was reported that *bisd*A and *bisd*B genes encoding ferredoxin and cytochrome P450 were responsible for BPA degradation in *Sphingomonas bisphenolicum* strain AO1 [25]. Most of cytochrome P450 (CYP_450_) monooxygenase system in bacteria are classified as type one (class I), which require NADH-dependent reductase (ferredoxin reductase) and an iron-sulfur redoxin (ferredoxin) for activation [26–28]. CYP_450_ monooxygenase systems are involved in the degradation of diverse xenobiotic compounds via catalyzing chemical reactions such as epoxidation, hydroxylation, dealkylation, and sulfoxidation [29].

*Pseudomonas putida* YC-AE1 was isolated from soil in our previous work, and its degradation capability towards BPA was evaluated. Promising strain YC-AE1 was able to degrade 200 mg l^−1^ of BPA within 20 h [21]. In order to elucidate the molecular mechanism underlying the degradation process, we employed transcriptome analysis to determine YC-AE1 strain-related genes that have potential implications in BPA degradation. Further, we investigated the role of CYP_450_ monooxygenase in BPA degradation. Our study opens new horizons for dealing with BPA and limiting its spread. Future studies are required to identify the rest steps in BPA degradation pathway by using those gene proposed in our study to elucidate the degradation pathways and its corresponding enzymes.

## Materials and methods

### Chemicals, media and strain

Bisphenol A was obtained from Shanghai Macklin Biochemical Co., Ltd. China (purity above 99%), other chemicals used in this study were high purity. Organic solvents were HPLC grade. The stock solution of BPA (20,000 mg l^−1^) was dissolved in methanol (99%), filtrated with specific filters then stored at 4 °C. Luria–Bertani (LB) medium was used to grow bacterial strains. Trace Elements Medium (TEM) was used for degradation assessment [30]. *Pseudomonas putida* YC-AE1 strain used in this study was isolated and identified in our previous work as a BPA degrader [21].

### Bacterial growth and samples preparation

To prepare the bacterial culture for transcriptome analysis, a fresh culture of *Pseudomonas putida* YC-AE1 (OD_600_ = 0.8) was inoculated in TEM supplemented with 100 mg l^−1^ BPA; the control culture (CK) was performed by adding 10% glucose as a sole carbon source without BPA. Bacterial cultures were grown at 30 °C in a rotary shaker (180 rpm) for 24 h. After incubation, the culture was taken in triplicates from each experiment and centrifuged at 15,000 rpm for 10 min. The pellet was collected and shocked with liquid nitrogen and kept at -80 °C.

### Library preparation and transcriptome sequencing

Total RNA extraction was performed from the above mentioned samples as described in the supplementary data. The library construction was prepared as descriped by Wang L et al. [31]. Briefly, ribosomal RNA (rRNA) was removed using biotin-labeled specific probes; the RNA was purified and fragmented. Illumina TruSeq was subsequently used. Random primers were used to synthesize fragmented cDNA, and the cDNA for these fragments was synthesized using the reverse transcriptase. DNA polymerase I and RNase H were used to synthesize a cDNA strand. dTTP was replaced with dUTP during the synthesis of the cDNA second strand. The double-stranded cDNA was then A-tailed and ligated to a sequencing adapter. The ligated product was purified and amplified to obtain the final cDNA library. Finally, the constructed sequencing library was sequenced using HiSeq sequencing platform.

### Data filtering and reference genome alignment

To clean up the raw reads (raw data), low-quality, linker contamination, and excessively high levels of unknown base N were filtered out (using in-house software). Also, Q20, Q30, GC content, and sequence duplication level of the clean data were calculated. To ensure the accuracy of subsequent analysis results, a short oligonucleotide alignment program (SOAP) [32] was used to compare reads to rRNA reference sequence. Filtered reads were saved in FASTQ format for subsequent analysis [33]. Hierarchical indexing for spliced alignment of transcripts (HISAT) [34], a fast and sensitive comparison software for RNA-seq reads, was used to compare the filtered clean reads to the reference genome. It has equal or better accuracy in RNA data than other methods [34].

### Gene expression level analysis

Bowtie2 software was employed to compare clean reads to reference genes [35], and then RNA-Seq by Expectation–Maximization (RSEM) [36] software package to calculate gene expression levels. The fragments per kilobase of transcript per million mapped reads (FPKM value) was used to quantitively evaluate gene expression. Based on the expression data, gene coverage reads distribution and sequencing saturation were evaluated and piloted using R software. The correlation of samples was done using the cor function in R software. Principal component analysis (PCA) was performed using the ade4 software package in R software. The clustering tree analysis was carried out using the hclust function in R software, with Euclidean as the distance algorithm.

### DEGs screening and enrichment analysis

DEGs were detected using the DESeq2 algorithm. The DESeq2 method is based on a negative binomial distribution model; then, a significance test was performed based on the estimated gene expression [37]. The calculated *p*-value was corrected by Bonferroni [38]. Based on the results of DEGs detection, a scatter plot of DEGs (Volcano plots) was plotted. KEGG can recognize the significant biochemical metabolic pathways and signal transduction network involved in significantly regulated genes [39]. Gene ontology (GO) analysis gives a function of significantly differently expressed genes compared to the background of the gene set. After Bonferroni corrected the calculated p-value, the corrected *p*-value is less than or equal to 0.05 as the threshold value. GO terms that meet this condition are defined as GO terms that are significantly enriched in differentially expressed genes. The GO function significance enrichment analysis can determine the major biological functions.

### qRT-PCR analysis

To validate the DEGs by qRT-PCR, YC-AE1 strain was cultured in TEM supplemented with 100 mg l^−1^ BPA in the treated culture and 10% glucose in the control culture. According to the manufacturer's instructions, total RNA was extracted from our strain YC-AE1 using RNAprep Pure Cell/Bacteria Kit (TIANGENE Biotech, Beijing CO., Ltd., China). TIANScript RT Kit (TIANGEN, China) was used for cDNA synthesis. Bio-Rad IQ5 PCR was used to perform the PCR reaction using an identical PCR condition. 16S rRNA gene served as an internal control. All primers used for qRT-PCR are listed in Table S1 in the supplementary material.

### CYP_450_ inhibition

To investigate whether CYP450 is implicated in BPA degradation by our strain, 1-aminobenzotriazole (ABT) was used as a CYP450 inhibitor [40]. TEM (10 ml) supplemented with different concentrations of ABT (final concentration, 0.1, 0.4, 1, and 2 mmol l^−1^) were inoculated with fresh broth culture of *Pseudomonas putida* YC-AE1. The cultures were incubated at 30 °C in a rotary shaker (180 rpm) for 2 h, followed by the addition of BPA (100 mg l^−1^), and further incubated for 24 h. HPLC measured the residuals of BPA [20]. The control culture was adjusted without adding ABT. All experiments were conducted in three independent replicates.

bisdB Knockout.

### *bisdB* knockout

For CYP450 (encoded by *bisdB* gene) knockout, pEX18TC-*bisdB* was constructed by fusing the purified PCR product of a kanamycin-resistant gene (kan), an upstream fragment of *bisdB* gene amplified with primers (bisdBuf-F and bisdBuf-R) and a downstream fragment amplified with primers (bisdBdf-F and bisdBdf-R) to *Hind* III and *Sac* I pre-digested pEX18TC plasmid using the Uni Seamless Cloning and Assembly Kit (Transgen). The recombinant vector (pEX18TC-bf-kan-af) was then transformed into *E. coli* Trans1-T1 by heat shock method. The purified recombinant plasmid was transformed to *E. coli* SM10 λpir before its conjugation with strain YC-AE1. The double-crossover recombinants of strain YC-AE1Δ*bisdB* were screened on LB plates containing 50 mgl^−1^ of Ampicillin (endogenous antibiotic resistance from *Pseudomonas putida* YC-AE1), kanamycin, and 15% (wt/vol) sucrose. The growing colonies were picked out and confirmed by 16S rRNA sequencing and running PCR for *bisdB* gene. To determine the effects of gene knockout, the strain of knockout mutants and wild type were cultured in a TEM medium with 100 mg l^−1^ BPA and incubated at 30 oC for 48 h in a rotary shaker at 185 rpm. One ml medium was filtered by an organic filter membrane (0.22 µm). Then the concentration of BPA was determined by an Agilent 1200 system HPLC (Agilent, USA) as described by Eltoukhy et al. [21]. The Agilent 1200 system HPLC (Agilent, USA) with visible ultraviolet detector was used equipped with Eclipse XDB C18 column (4.6 × 150 mm × 5 μm). The mobile phase was acetonitrile 85% and (water, 0.1% acetic acid) 15%, with a flow rate 1 ml/minute and detecting wave length 220 nm. Strains, plasmids and primers used in knocking out of *bisd B* are described in Table 1.

**Table 1:** Strains, plasmids and primers used in cloning and expression of *bisd A* and *bisd B* and knocking out of *bisd B*

Strains, Plasmids and Primers	Description	Source
***Pseudomonas putida* strains**		
YC-AE1	Wild-type bisphenol A degrader	[21]
YC-AE1Δ*bisdB*	YC-AE1 mutant with *bisdB* gene replaced with kanamycin resistance gene	This study
***E. coli* strains**		
Trans1-T1	F-φ80(lacZ)ΔM15ΔlacX74hsdR(r_k_^−^,mk^+^)Δ*rec*A1398endA1tonA	TransGen
BL21(DE3)	Host strain for expression vextors; F^−^ ompT hsdSB(r_B_^−^ m_B_^−^) gal *dcm*(DE3)	Tiangen
SM10λpir	Donor strain for conjugation, thi thr leu tonA lacY supE recA∷RP4-2-Tc∷Mu	Zomanbio
**Plasmids**		
pET32a( +)	Expression vector; Amp^r^	Novagen
pEX18Tc	Gene knockout vector, oriT, sacB, Tc^r^	Miaolingbio
pET32a-*bisdB*	pET-28a( +) derivative carrying *bisdB*	this study
pET32a-*bisdAB*	pET-28a( +) derivative carrying *bisdA* and *bisdB*	this study
pEX18Tc-bisdB	pEX18Tc derivative carrying *bisdB*	this study
**Primers Sequence (5’ → 3’)**		
bisdB-F	GCGCGAGCTCATGAACCCTCAGACACTGC	this study
bisdB-R	GCGCAAGCTTGTTTTTGTCCCAGACCAGC	this study
bisdAB-F	GCGCGAGCTCATGCCTCATATCCAAGTGACT	this study
bisdAB-R	GCGCAAGCTTGTTTTTGTCCCAGACCAGC	this study
bisdBup-F	TAAAACGACGGCCAGTGCCATTACTCAGCGAGCCGCGTT	this study
bisdBup-R	TCCCGTTGAATATGGCTCATGTTCGGATTCCCGCTCATTTTCG	this study
kan-F	ATGAGCCATATTCAACGGGAAACGT	this study
kan-R	TTAGAAAAACTCATCGAGCATCAAATGAAAC	this study
bisdBdown-F	GATGCTCGATGAGTTTTTCTAAGCCGGGCTTTCAAGTACCTGAGCAGATG	this study
bisdBdown-R	ACCATGATTACGAATTCGAGCTGCCATCGACTGCGCAGACATG	this study

*Amp*^*r*^ Ampicillin resistant, *Tc*^*r*^ Tetracycline resistant; the restriction sites in the primers (5’ → 3’) are underlined

### Cloning and Expression of *bisdB* and *bisdAB*

DNA from *Pseudomonas putida* YC-AE1 was extracted using Bacterial Genomic Extraction Kit (Takara, Japan). Gene *bisdB* (CYP450) was amplified by using bisdB-F, bisdB-R primers, while *bisdAB* (ferredoxin and CYP450) was amplified by bisdAB-F and bisdAB-R (Table 1). The PCR products of *bisdB* and *bisdAB* were purified using DNA fragment purification kit (Takara, Japan). The purified products were ligated to the digested pET32a( +) vector to obtain the recombinant plasmids pET-32a-*bisdB* and pET-32a-*bisdAB*. Two recombinant plasmids were transformed separately into *E. coli* BL21(DE3) by heat shock method. To detect the ability of transformed cells to degrade BPA, the recombinants *E. coli* BL21(DE3) were grown in L.B medium supplemented with BPA (100 mg l^−1^) until (O.D 0.8) followed by induction with Isopropyl β- d-1-thiogalactopyranoside (IPTG) at a final concentration of 1 mmol. BPA degradation was detected using HPLC, wild type *E. coli* BL21(DE3) with an empty vector was used as control.

### Accession numbers

The gene sequences of both *bisdA* and *bisdB* were submitted in the Genbank database with accession numbers MW113668 and MW113669, respectively. The whole-genome sequence of *Pseudomonas putida* strain YC-AE1 was deposited in Genbank with Bioproject ID, PRJNA597651 and reference NZ_CP047311.1, NZ_CP047312.1, NZ_CP047313.1, NZ_CP047314.1 for chromosome, plasmid 1, plasmid 2 and plasmid 3, respectively.

## Results and discussion

### General statistics of Pseudomonas putida YC-AE1 whole genome

The whole genome of *Pseudomonas putida* YC-AE1 consists of a circular chromosome and three plasmids with a total of 6,992,587 bp and GC content of 60.62%. The size of chromosome is 6,053,381 bp with GC content of 61.29% while, the 3 plasmids size was 504,084 bp, 388,915 bp and 46,207 bp with GC content of 56.28%, 56.09% and 57.73%, respectively. We identified Eighty-two copies of tRNA and twenty-two copies of rRNA in the bacterial genome.

### RNA-seq analysis

The total clean reads of control samples (CK) were 16.1, 14.89, and 16.07 Mb obtained from CK1, CK2, and CK3, respectively, with an average of 15.68 Mb and 90% clean reads. On the other hand, the total clean reads of treated samples (T) were 15.90, 15.82, and 14.74 Mb for T1, T2, and T3, respectively, with an average of 15.48 Mb 88.92% clean reads ratio. The Q20 and Q30 of all samples were more than 98 and 95%, respectively, indicating the high quality of clean reads [41], (Table S2). The clean reads were mapped onto the genomic sequences of *Pseudomonas putida* YC-AE1, with high mapping rates, an average of 96% for control and 95.75% for treated groups. The new transcript prediction analysis yielded 1405 new transcripts, with 107 and 1298 for coding and non-coding transcript, respectively. The new transcript prediction analysis yielded 1405 new transcripts, most of them representing the non-coding region by 1298 reads, in which 710 for intergenic region and 588 for antisense to mRNA, coding region was represented by 107, calculated as 63 for intergenic region and 44 for antisense to mRNA (Table S3). Gene expression level was determined based on the expected number of FPKM attributed to gene numbers. As shown in Fig. S1, in FPKM <  = 100, the average of gene numbers expressed in control and treated were 1523 and 1662, respectively, while, in FPKM >  = 100, the expressed gene numbers were 1884 and 1635 on average for control and treated samples, respectively. The largest gene numbers were expressed in FPKM between 10–100, approximately 2558 and 2666 on average for control and treated samples, respectively.

To evaluate the degree of similarity between samples, the correlation and variance were calculated. In this study, a principal component analysis (PCA) and a heat map were performed to visualize this correlation. As depicted in the heat map and PCA (Fig. S2 and S3), the correlation between control and treated samples was high, which indicates the correctness of the sampling.

### Identification and analysis of DEGs

The expression level of 6165 genes identified from RNA-seq between control and treated group were quantified by FPKM and folding change **|**Log2FC**|** with DEGseq2 into three groups. There was a total of 725 genes with an expression level at **|**Log2FC**|≥ **1 and Padj ≤ 0.05 were identified as significantly up-regulated genes [41], while 504 genes at expression level **|**Log2FC**|**≤ -1 and Padj ≤ 0.05 were identified as significantly down-regulated, while, 4936 genes were regarded as non-significantly regulated at expression level abs**|**Log2FC**|**< 1 and Padj > 0.05. It is plausible that, BPA degradation related genes are up-regulated, while the down-regulated genes may reflex the response of *Pseudomonas putida* YC-AE1 to toxic effects upon exposure to BPA. [42], reported a total of 839 genes as DEGs in response to biodegradation of chlorimuron-ethyl by *Klebsiella jilinsis* 2N3 with 386 up-regulated and 453 down-regulated.

**Fig. 1:**
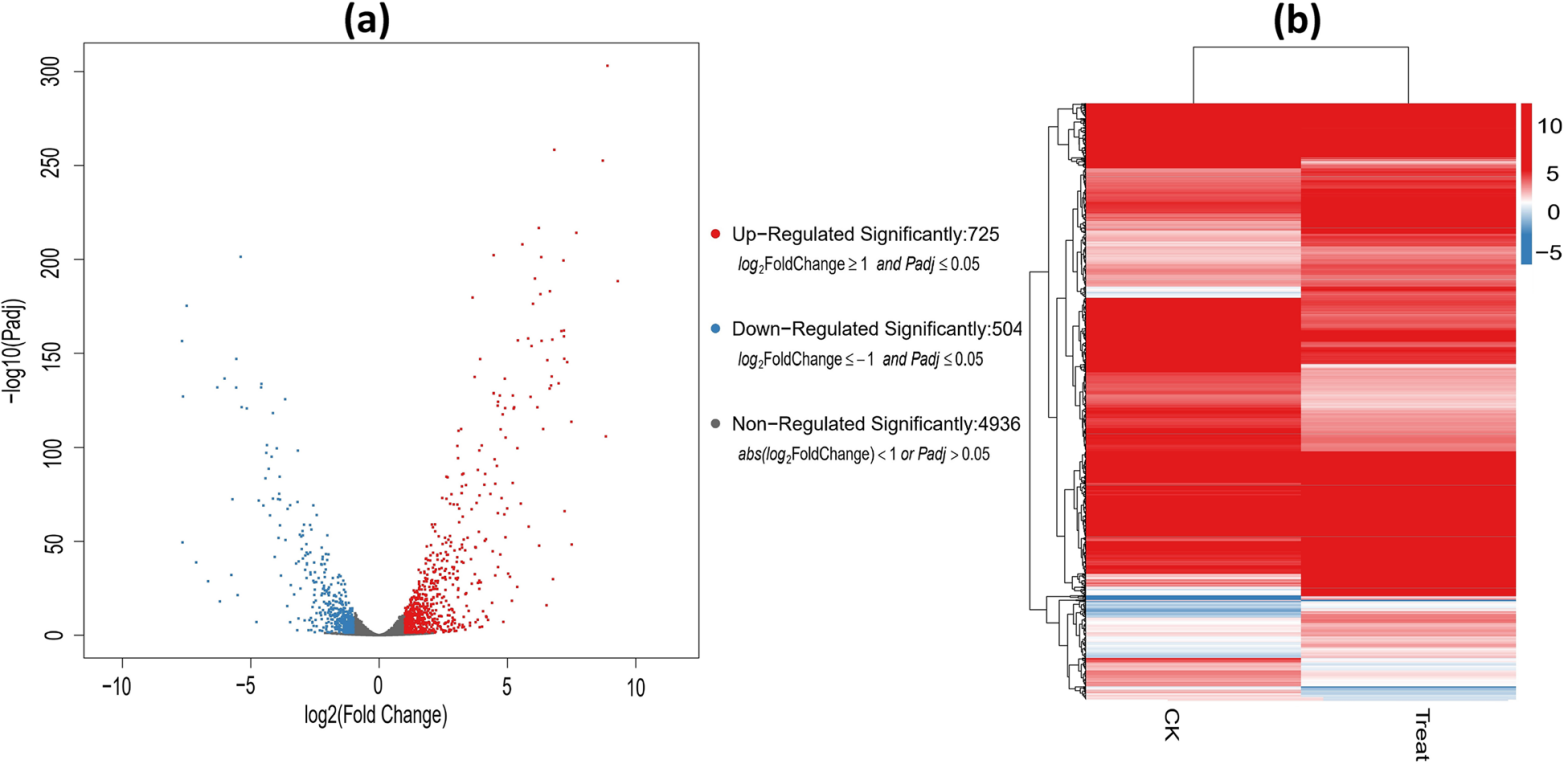
Expression and analysis of DEGs (**a**), volcano map of DEGs during BPA degradation showing the expression fold change (X axis) and significant value, representing the up-regulated (red), down-regulated (blue), and non-regulated genes (black), (**b**) heat map of significantly DEGs during BPA degradation with cluster analysis. The horizontal axis represents the samples for cluster analysis, and the vertical axis represents significant DEGs. The color represents the expression level after logarithmic conversion; the color difference implies the variation in gene expression

A volcano plot illustrated in Fig. 1a, which can quickly screen out genes with significant statistical differences, was used to visualize these data. The horizontal axis represents the value of the multiple of the difference after log2 conversion, and the vertical axis represents the significance value after -log 10 conversion. A higher position means that the gene has a smaller *p*-value in the differential pair. According to the color of the legend, it is divided into significantly up-regulated DEGs, significantly down-regulated DEGs, and non-regulated DEGs. A heat map was created to visualize the clusters of significantly DEGs between the control and treated groups, as shown in Fig. 1b. The heat map was adopted relying on FPKM of RNA-seq data and represented the genes expression levels. DEGs were classified in the bar according to the log10 (FPKM + 1) value.

### Functional enrichment analysis of DEGs in *Pseudomonas putida* YC-AE1

**Fig. 2:**
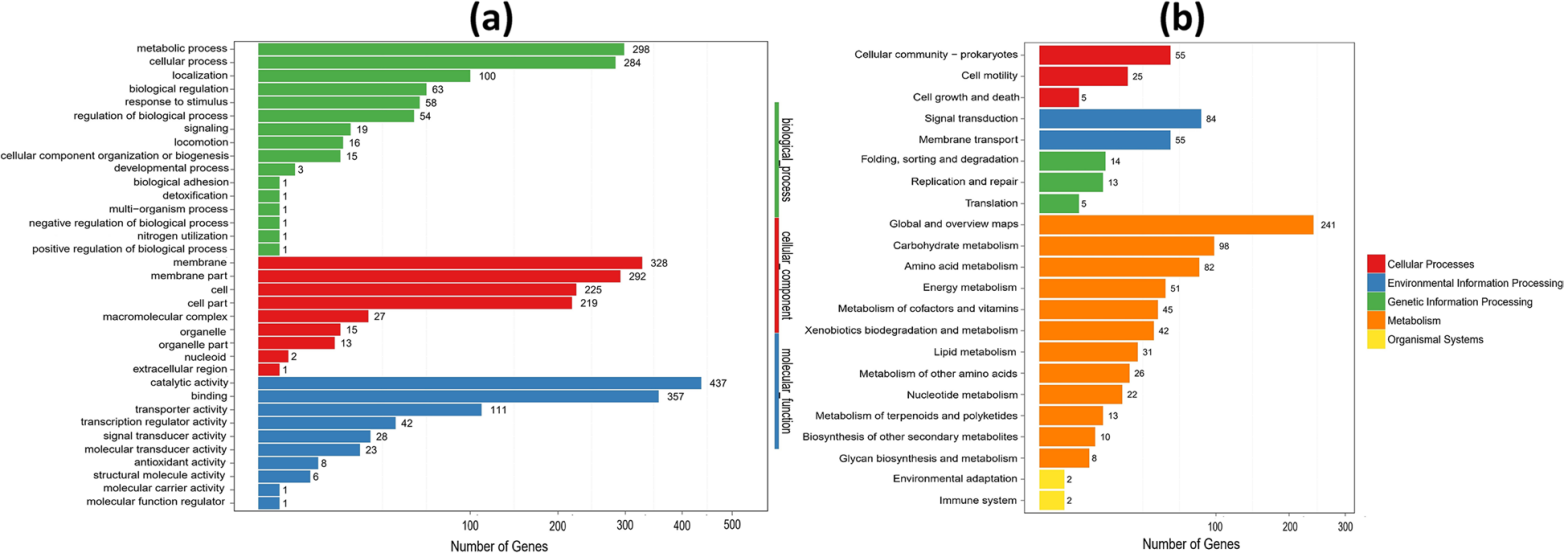
**(a)** Classification of GO functions of significant DEGs. The abscissa represents the number of significant DEGs, and the ordinate represents GO terms. There are three categories of GO terms, which are marked with different colors, (**b**) Classification diagram of KEGG Pathway of significant DEGs [44]. The abscissa is the number of significantly DEGs, and the ordinate represents the pathway classification, which is marked with different colors according to the annotations

Gene Ontology is an international classification system to unify and describe the gene and gene product attributes in organisms [43]. GO describes the molecular function, cellular component, and biological processes. All DEGs were categorized into the three terms in which biological process with 916 (30.1%), cellular component 112 (36.5%), and molecular function 1014 (33.3%) (Fig. 2a). DEGs in response to BPA degradation by YC-AE1 were associated mainly with the metabolic and cellular processes represented by 298 and 284 genes, respectively. In the context of molecular function, catalytic activity and binding were mapped with the highest number of DEGs, 437 and 357 genes, respectively. Altogether, these data indicated that the metabolic functions of *Pseudomonas putida* YC-AE1 were affected upon exposure to BPA, and the DEGs of these GO terms may be linked to the metabolizing process of BPA by YC-AE1 strain.

The pathway-based analysis is crucial to elucidate the biological functions of genes. To further analyze the potential role of DEGs, we performed a KEGG pathway analysis to identify the metabolic pathways enriched in the BPA degradation process [45]. Results showed that the 135 different metabolic pathways between CK and T were involved in five categories, cellular process (CP), environmental information processing (EIP), genetic information processing (GIP), metabolism (M), and organismal systems (OS). A total of 929 DEGs were involved in the pathways with 669 genes (72%) in M category, followed by EIP with 139 genes (~ 15%) (Fig. 2b).

**Fig. 3:**
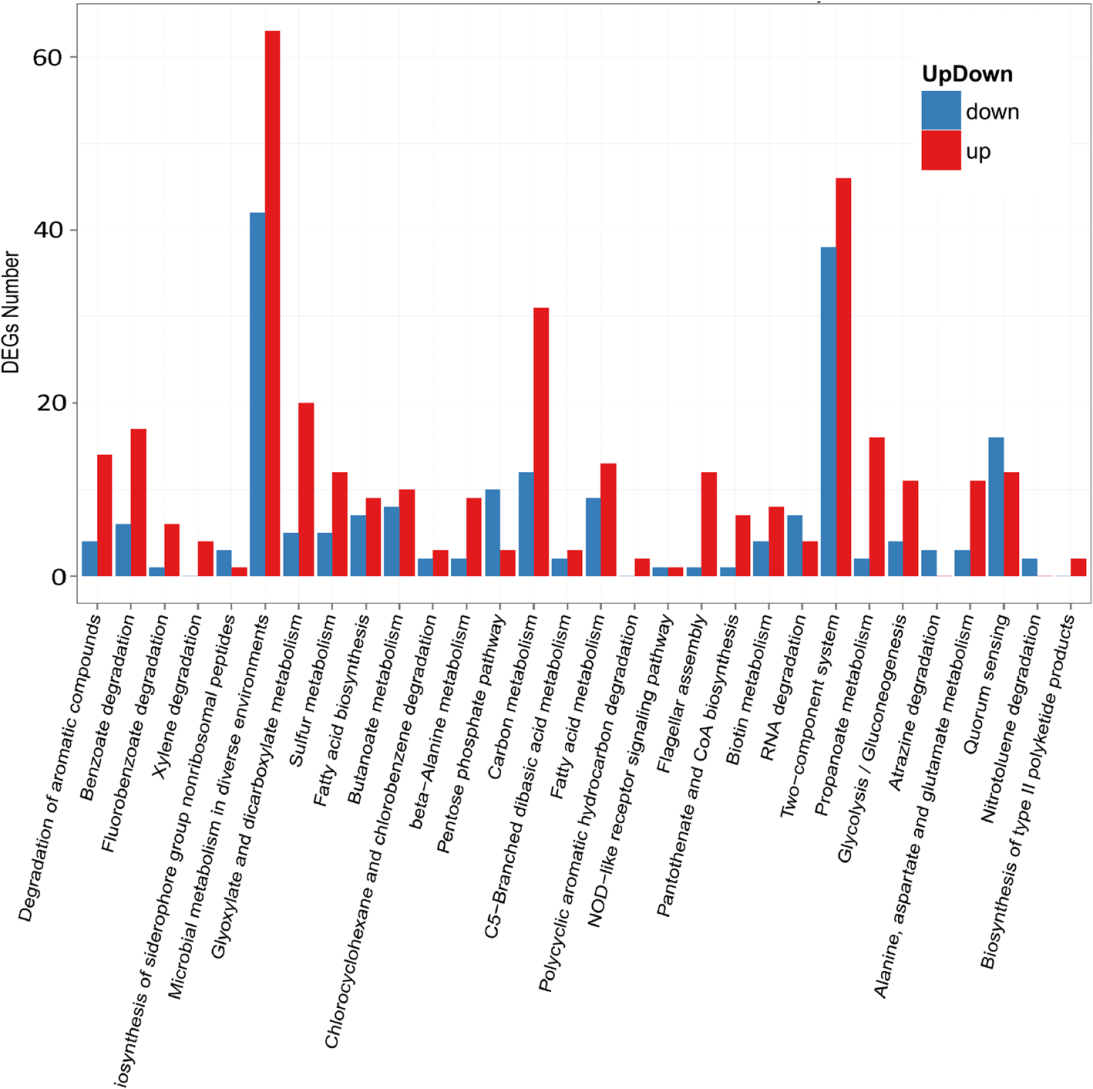
Pathway classification map of significantly enriched DEGs. The abscissa is the pathway for the enrichment of significantly DEGs (arranged by the degree of enrichment significance, and the significance of enrichment decreases from left to right), and the ordinate indicates the number of significantly up-regulated DEGs in the corresponding pathway

The numbers of regulated genes were illustrated in Fig. 3; the highest number of up and down-regulated genes belong to microbial metabolism in diverse environments pathway with 63 and 41 DEGs, respectively, followed by two-component system pathway and carbon metabolism pathways with 86 and 43 DEGs. Efflux pump related genes have been reported to play a crucial role in microbial environmental adaptability, for instance, SrpABC from *Pseudomonas putida* strain B6-2 (DSM 28,064) enhanced the degradation capacity toward polycyclic aromatic hydrocarbon (PAH) via extruding toxic intermediates during the degradation [46]. In contrast, EmhABC pump reduced the degradation capability of *Pseudomonas fluorescens* strain LP6 via excreting out its substrate [47]. TtgABC efflux machinery enhances phenol tolerance of *Pseudomonas putida* strain DOT-T1E because this pump contributes to toluene tolerance [48, 49]. Adaptation of bacterial cell to environmental pollutants and orchestrating their gene response accordingly largely reside on two-component signal systems (TCS) [50]. *Pseudomonas putida* ColR (TCS) deleted strain showed attenuation in phenol tolerance compared with wild type [51]. In our study, thirty efflux pump related genes were regulated; in which 12 genes were positively regulated and 18 genes were negatively regulated. In the same context, 193 genes related to two-component system have regulated, 86 up regulated and 107 down regulated. However their specific roles in PBA degradation still unknown and required further investigation.

**Fig. 4:**
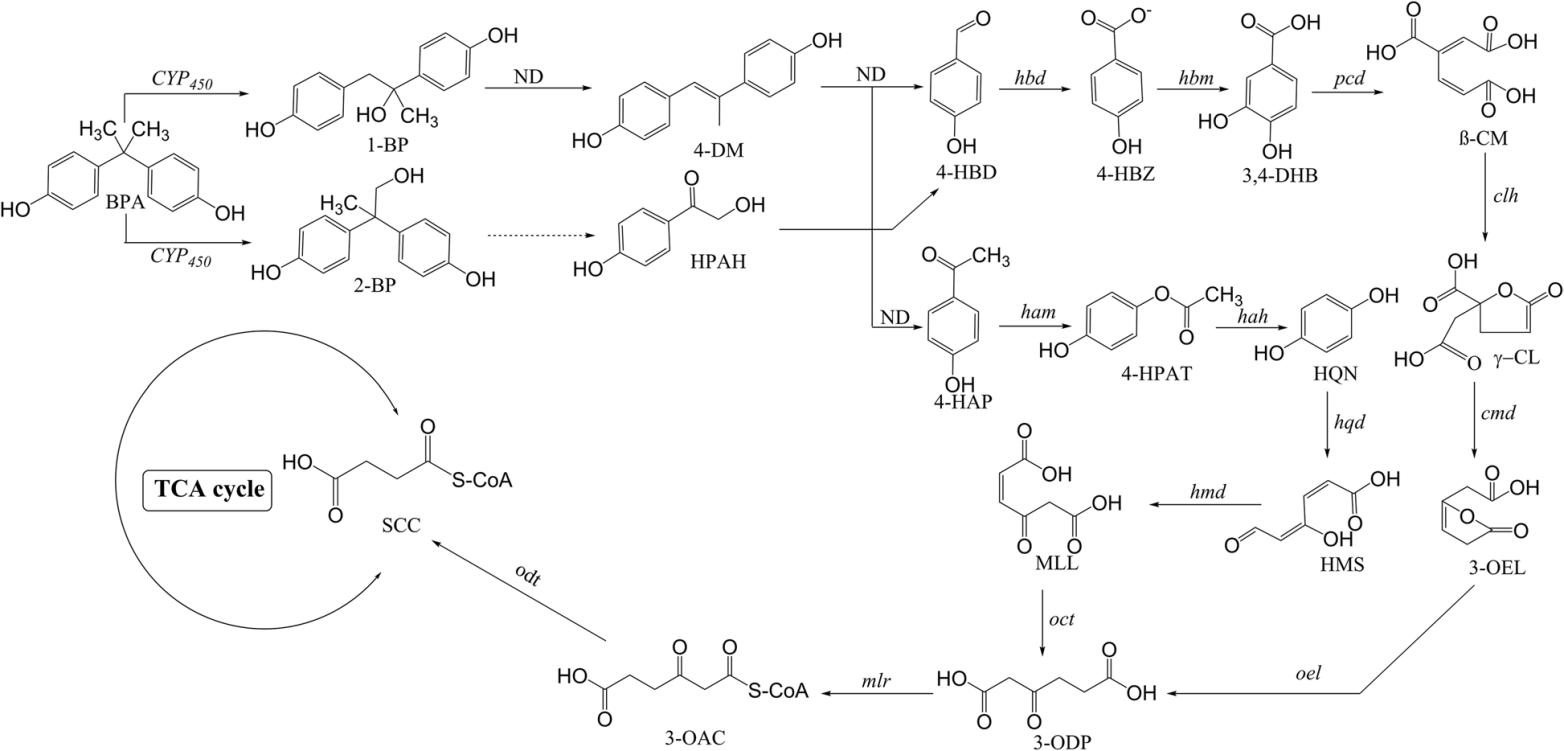
BPA biodegradation pathways by *Pseudomonas putida* YC-AE1 and recommended enzymes involved in the mineralization. Intermediates marked by orange indicate the degradation products detected by HPLC/MS in our previous study [21]. Abbreviations, BPA, bisphenol A; 1-BP, 1,2-bis(4-hydroxyphenyl)-2-propanol; 2-BP, 2,2-bis(4-hydroxyphenyl)-1-propanol; 4-DM, 4,4′-dihydroxyl-α-methylstilbene; 4-HPAH, 4-hydroxyphenacyl alcohol; 4-HBD, 4-hydroxybenzaldehyde; 4-HBZ, 4-hydroxybenzoate; 4-HAP, 4-hydroxy-acetophenone; 4-HPAT, 4′-hydroxyphenyl acetate; HQN, hydroquinone; 3,4- DHB, 3,4-dihydroxybenzoate; HMS, 4-hydroxymuconic semialdehyde; MLL, maleylacetate; β-CM, β-carboxy-muconate; γ-CL, gamma-carboxymucono-lactone; 3- OEL, 3-oxoadipate-enol-lactone; 3-ODP, 3-oxoadipate; 3-OAC, 3-oxoadipyl-CoA; SCA, succinyl-CoA. Gene names are summarized in Table S4 in Supplementary Material

According to the intermediate compounds and the proposed pathway of BPA degradation by YC-AE1 in our previous study [21], accompanied by data generated from transcriptome analysis, we hypothesized that 15 genes could be involved in BPA metabolism. The up-regulated genes in the degradation process are probably associated with BPA metabolism or the improvement of BPA tolerance in YC-AE1 strain. Overall, the mRNA expression levels of the investigated 15 genes were significantly higher than the control group. The information about the regulated genes, including name, ID, and EC number for encoding enzymes, are summarized in Table S4 in the Supplementary Material. The degradation pathways of BPA by YC-AE1 obtained from transcriptome analysis and pathway enrichment are illustrated in Fig. 4. As shown in Fig. 4, CYP450 monooxygenase (Gene ID: YCAE1GL005919) was proposed to initiate the first step in BPA degradation in which BPA is transformed to 1,2-bis(4-hydroxyphenyl)-2-propanol and/or 2,2-bis(4-hydroxyphenyl)-1-propanol, this finding was consistent with the previous report by Sasaki et al. [52]. CYP450 monooxygenases are a superfamily of ubiquitous heme- monooxygenases that participate in the microbial degradation of diverse materials such as oils, fuel additives, and chlorinated hydrocarbons [53]. Cheng et al. [54] revealed the potential role of CYP450 in the biodegradation of Chlorimuron-ethyl by *Rhodococcus erythropolis* D310-1. Moreover, the CYP450 mRNA level was significantly up-regulated in the aminobenzoate degradation pathway, inconsistent with the production of the degradation products [42].

Our results also showed that the dehydratase enzymes were significantly up-regulated. These enzymes are involved in the transformation of 1,2-bis(4-hydroxyphenyl)-2-propanol to 4,4-dihydroxy-alpha-methylstilbene which is further converted to p-hydroxyacetophenone and p-hydroxybenzaldehyde. To detect whether YC-AE1 can further metabolize p-HBAL and p-HAP, a TEM medium supplemented with 100 mg l^−1^ p-HBAL and p-HAP as a sole carbon source was inoculated with YC-AE1 and incubated at 30 oC for 24 h. YC-AE1 strain showed 100% degradation capacity of p-HBAL and p-HAP in 24 h (data not shown); this is evidence that YC-AE1 strain could mineralize p-HBAL and p-HAP. Our finding can interpret the high mineralization rate for BPA by YC-AE1 strain in our previous study [21]. Also, our RNA seq data showes significant upregulation to oxidoreductase related genes.

The activity of oxidoreductase act on CH-CH group and CH-OH group [55]. Twenty-one up-regulated genes were detected in our transcriptome with an expression level at **|**Log2FC**|≥ **1 and Padj ≤ 0.05. Our results indicated that *Pseudomonas* putida YC-AE1 over-expressed oxidoreductase encoding genes to improve the biodegradation of BPA oxidation and to form hydroxylated benzene rings of BPA. Our data was compatible with data reported by wang et al. [55] in BPA biodegradation by the green alga *Desmodesmus* sp.WR1. García-Rodríguez et al. [56] reported that phenolic compounds could be oxidized by oxidoreductases like polyphenol oxidase and peroxidase.

### qRT-PCR Verification

**Fig. 5:**
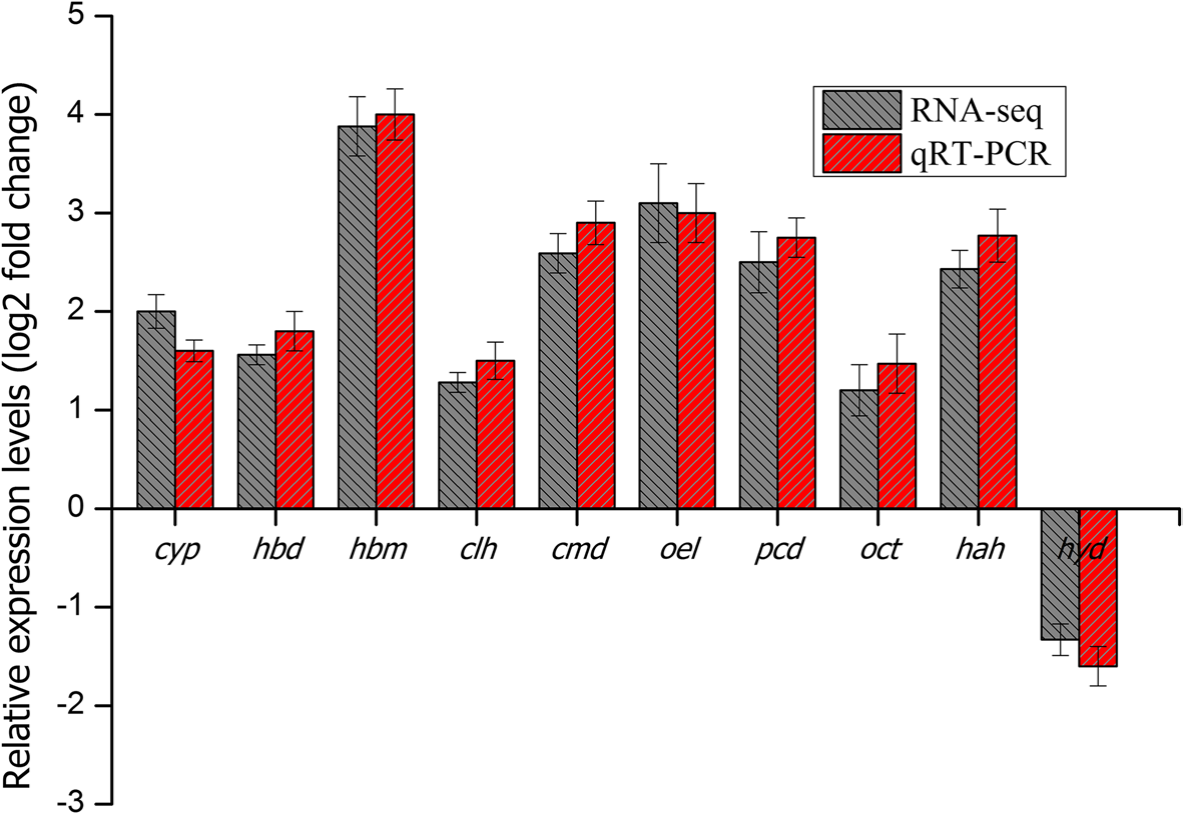
Validation of RNA-seq data by qRT-PCR. Ten DEGs involved in the BPA degradation by YC-AE1 were selected for verification. The data were expressed as the mean fold change relative to the control samples

Ten genes involved in the BPA degradation pathway were selected for further verification using qRT-PCR analysis. Genes were abbreviated as follows: CYP450 monooxygenase (cyp), 4-hydroxybenzaldehyde dehydrogenase (hbd), 4-hydroxybenzoate 3-monooxygenase (hbm), γ-carboxymucono-lactone hydrolase (clh), 4-carboxymuconolactone decarboxylase (cmd), 3-oxoadipate enol-lactonase (oel), protocatechuate 3,4-dioxygenase (pcd), 3-oxoadipate CoA-transferase (oct), 4-hydroxyacetophenone hydrolase (hah) and hydroxyquinol 1,2-dioxygenase (hyd). Figure 5 showed the relative transcription level of selected genes in both RNA-seq data and qRT-PCR. The result was consistent with RNA-seq data and confirmed the upregulation of the BPA degradation genes in the YC-AE1 strain.

### *CYP*_*450*_* mediates BPA degradation in YC-AE1*

**Fig. 6:**
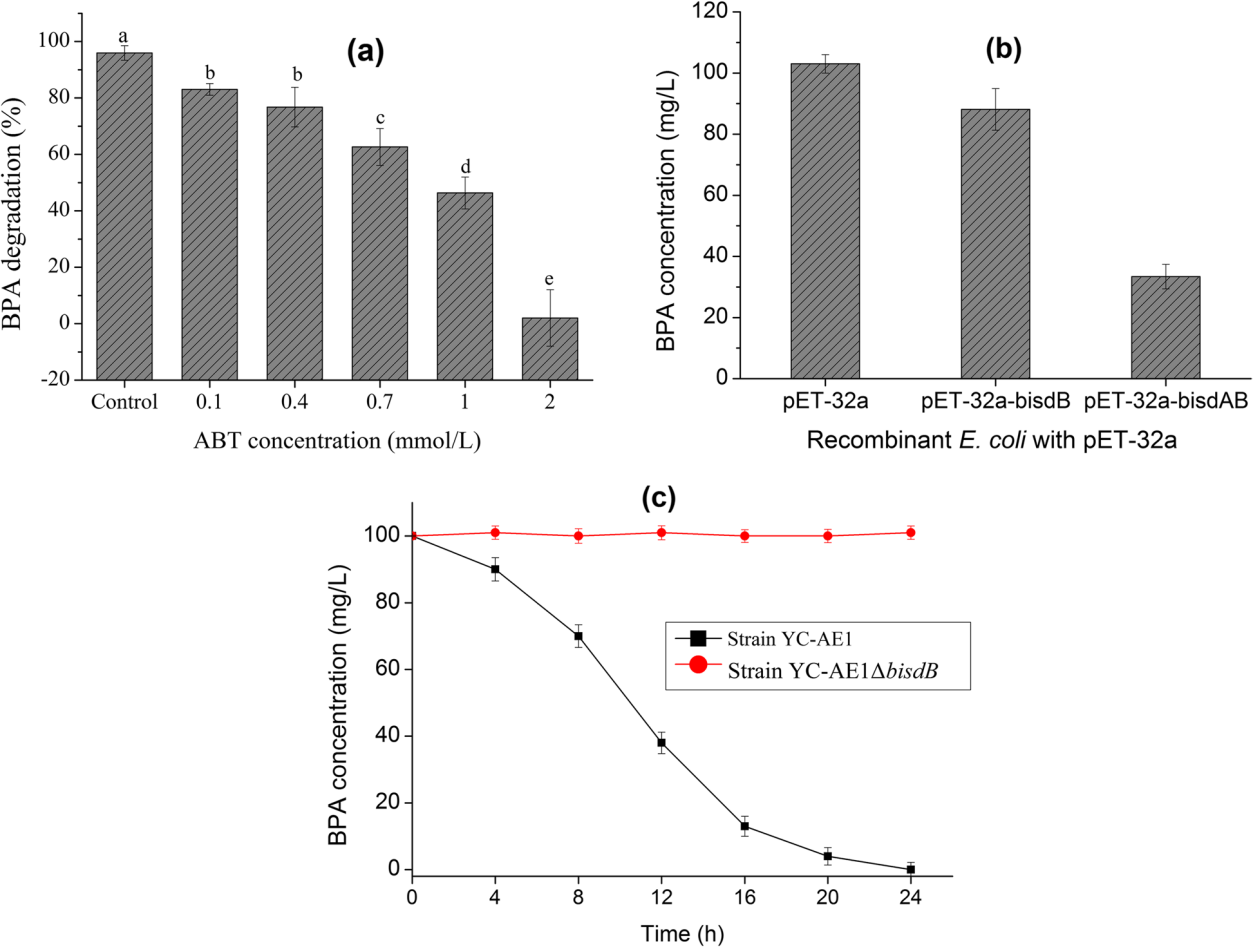
**a** Effect of CYP_450_ inhibitor ABT on BPA degradation by *Pseudomonas putida* YC-AE1 after 24 h of incubation, (**b**) BPA degradation by *bisdB*- and *bisdAB*- recombinant *E. coli* cells after 24 h incubation in LB medium. *E.coli* cells with empty pET-32a were used as control, (**c**) BPA degradation by strain YC-AE1 and Knocked strain YC-AE1ΔbisdB in TEM medium

To confirm the involvement of CYP450 in BPA degradation by YC-AE1, we used ABT, which is known as an inhibitor for CYP. The addition of 0.1, 0.4, 0.7, 1, and 2 mmol of ABT significantly decreased the efficiency of YC-AE1 to degrade BPA to 83, 76.7, 62.6, 46, and 2%, respectively, compared to 95% BPA degradation in control, Fig. 6a. These findings indicated that CYP is involved in BPA catabolism, consistent with the previous studies that reported the involvement of CYP in the hydrolysis of many xenobiotic compounds [57, 58]. Mtibaà et al. [57] Reported a slight decrease in BPA removal rate in the presence of ABT as a CYP450 inhibitor. Further, Wei et al. [58] reported a 55% decrease in triphenyl phosphate (TPHP) degradation by *Bacillus* *brevis* in the presence of 1 mmol of piperonyl butoxide (PB) as a CYP450 inhibitor. Sasaki et al. [59] reported that CYP encoded by *bisd* in *Sphingomonas bisphenolicum* AO1 is responsible for the transformation of BPA. This finding indicates the indispensable role of CYP450 as an essential initiator during BPA degradation in YC-AE1 strain. *E. coli* genome encodes ferredoxins and ferredoxin reductases, indicating that *E. coli* cells bearing *bisdB* might degrade BPA [25]. We cloned the *bisdA* and *bisdB* genes from YC-AE1 in *E. coli* BL21 (DE3). As shown in Fig. 6b, the recombinant *E.coli* carrying pET-32a-*bisdB* could degrade 12 mg l^−1^ of BPA after 24 h of incubation, while *E.coli* harboring pET-32a-*bisdAB* possess the ability to degrade about 66 mg l^−1^ after 24 h incubation. Ferredoxin of *Pseudomonas putida* YC-AE1 was implicated in BPA degradation by CYP*bisd*, and that can be seen obviously from the high difference in BPA degradation rate in *E.coli* harboring only *bisdB*, and that contain *bisdAB*. Together, these results suggested that the ferredoxins of *E. coli* could not meet the requirement well enough for a high degradation rate of BPA [20].

### Knockout of *bisdB* in YC-AE1

The previously mentioned data in this study provided evidence that CYP*bisd* is involved in BPA degradation by YC-AE1; however, this is not reliable evidence that no other genes in YC-AE1 can perform the same function. Thus, it is necessary to test whether *bisdB* deleted YC-AE1 could metabolize BPA. Therefore, *bisdB* YC-AE1 knockout strain was constructed and tested for BPA degradation. Interestingly, YC-AE1Δ*bisdB* completely lost its ability to degrade BPA and failed to grow on BPA as a sole source of carbon and energy, Fig. 6c. This implied that *bisdB* gene is indispensable for initiating BPA catabolism in our strain. In the same context, Zhang et al. [42] reported the role of CYP encoded by Kj-CysJ in the biodegradation of Chlorimuron-ethyl by *Klebsiella jilinsis* 2N3, 2N3 deleted Kj-CysJ showed a lower degradation rate compared to wild type and was no longer able to utilize Chlorimuron-ethyl as carbon source. This finding, coupled with those of qRT-PCR and transcriptomic analysis, confirmed that CYP450 was a key enzyme in the biodegradation process of BPA in YC-AE1 strain.

**Fig. 7:**
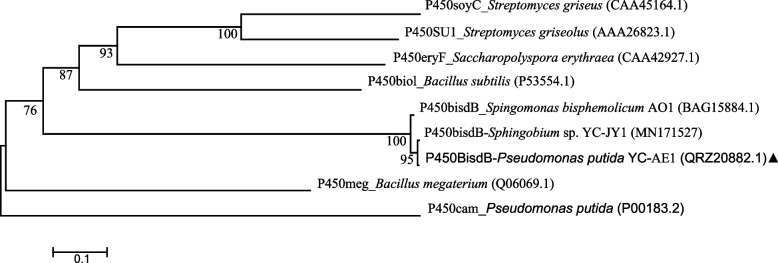
Phylogenetic analysis of P450_*bisdB*_ from strain YC-AE1 and other bacterial CYP_450_ proteins. The phylogenetic tree was constructed by the neighbor-joining method using MEGA 5.2 software. P450_*bisdB*_ from *Pseudomonas putida* YC-AE1 was marked using a dark triangle. The accession numbers of the protein sequences were in brackets

Phylogenetic analysis of P450 encoded by YC-AE1 *bisdB* and related bacterial CYP450s are depicted in Fig. 7. P450*bisdB* was clustered with P450*bisdB* from *Sphingobium* sp. YC-JY1 showed high similarity (99.5%) and 98.3% with P450 from *Sphingomonas bisphenolicum* AO1. P450*bisdB* showed little similarity with other P450s. The entire sequence of amino acids for BisdA and BisdB of YC-AE1 strain were shown in the supplementary data.

## Conclusion

Here, we unravel the molecular mechanism behind BPA degradation by *Pseudomonas putida* YC-AE1. Transcriptome analysis revealed the association of microbial metabolism pathway upon BPA stress. The biodegradation pathway was proposed with recommended involved genes and enzymes based on the functional annotation and gene expression profile during the degradation. The CYP450 encoded by *bisd* was introduced as an indispensable key gene in the biodegradation process of BPA by our strain using cytochrome inhibitor and gene knockout technology. More deep studies would provide evidence to verify further the functions of these genes identified in our research in BPA biodegradation.

## Supplementary Information

**Additional file 1: Table S1. **Primers sequences used in qRT-PCR. **Table S2.** Filtered reads quality statistics. **Table S3.** Statistics of New Transcript Types. **Table S4.** Summary of genes encoding enzymes involved in conversion of bisphenol A degradation products. **Fig. S1.** The distribution of gene expression in control (CK) andtreated (T) samples shows the number of expressed genes in each sample with a corresponding FPKM value. **Fig. S2.**Heat map of correlation between samples. **Fig. S3. **PCA analysis results for control and treated samples. **Fig. S4. **Phylogenetic tree for BisdA gene of *Pseudomonase putida* strain YC-AE1 and other related BisdA genes from other bacterial strains.

## Data Availability

All data generated or analysed during this study are included in this published article and its supplementary information file except, the gene sequences of *bisdA* and *bisdB* genes were submitted in the Genbank database with accession numbers MW113668 and MW113669, respectively and The whole-genome sequence of *Pseudomonas putida* strain YC-AE1 was deposited in Genbank with Bioproject ID, PRJNA597651.

## Abbreviations

BPA: Bisphenol A
DEGs: Differentially Expressed Genes
CYP: Cytochrome P; LB: Luria–Bertani
TEM: Trace elements medium
SOAP: Short oligonucleotide alignment program
HISAT: Hierarchical indexing for spliced alignment of transcripts; RSEM: RNA-Seq by Expectation–Maximization
GO: Gene ontology
ABT: 1-Aminobenzotriazole
kan: Kanamycin
PCA: Principal component analysis
CP: Cellular process
EIP: Environmental information processing
GIP: Genetic information processing
OS: Organismal systems
