# Supplementary material for: Transcriptome analysis and cytochrome P450 monooxygenase reveal the molecular mechanism of Bisphenol A degradation by Pseudomonas putida strain YC-AE1
Source: BMC Microbiol. 2022 Dec 9;22:294. doi: 10.1186/s12866-022-02689-6 (PMC9733184; doi:10.1186/s12866-022-02689-6)
Supplement: Supplementary file 1 — Additional file 1: Table S1. Primers sequences used in qRT-PCR. Table S2. Filtered reads quality statistics. Table S3. Statistics of New Transcript Types. Table S4. Summary of genes encoding enzymes involved in conversion of bisphenol A degradation products. Fig. S1. The distribution of gene expression in control (CK) andtreated (T) samples shows the number of expressed genes in each sample with a corresponding FPKM value. Fig. S2.Heat map of correlation between samples. Fig. S3. PCA analysis results for control and treated samples. Fig. S4. Phylogenetic tree for BisdA gene of Pseudomonase putida strain YC-AE1 and other related BisdA genes from other bacterial strains. [file 12866_2022_2689_MOESM1_ESM.docx]

|  |  | **Table S1.** Primers sequences used in qRT-PCR | | | |  |
| --- | --- | --- | --- | --- | --- | --- |
|  |  | |  | **Primers** | | |
| **Gene ID** | **Recommended name** | | **Abbrevation** | **Forward** | **Reverse** | |
| 16S rRNA | 16S rRNA | | 16S rRNA | 5’-CATGCTGATCTACGATTACT-3’ | 5’-CCATAAAGTTGTTCTCAGTT-3’ | |
| YCAE1GL005919 | Cytochrome p450 monoxygenase | | *cyp* | 5’-ATGAACCCTCAGACACTGCCGG-3’ | 5’-AATTCAGCGGCTTATAGAAGTGCAGC-3’ | |
| YCAE1GL000629 | 4-hydroxybenzaldehyde dehydrogenase | | *hbd* | 5’-ATGAGCATTGTTCAGCACCTGATTCA-3’ | 5’-CCTGCACGGTTGCGCGG-3’ | |
| YCAE1GL003062 | 4-hydroxybenzoate 3-monooxygenase | | *hbm* | 5’-ATGCCGCCCCGCTGCC-3’ | 5’-TACCAGCCTTGTGCAGCAGCTGG-3’ | |
| YCAE1GL004792 | Protocatechuate 3,4-dioxygenase | | *pcd* | 5’-ATGCCAATCGAACTGCTGCCG-3’ | 5’-CTGGCTTGGCCAGGCGGTT-3’ | |
| YCAE1GL004616 | γ-carboxymucono-lactone hydrolase | | *clh* | 5’-ATGAGCAACCAACTGTTCGACGC-3’ | 5’-AACCGGCAGAGGCTTCGGC-3’ | |
| YCAE1GL004614 | 4-carboxymuconolactone decarboxylase | | *cmd* | 5’-ATGGACGAGAAACAACGTTACGACG-3’ | 5’-GGCGGGTGATCATTTCCTGGA-3’ | |
| YCAE1GL004615 | 3-oxoadipate enol-lactonase | | *oel* | 5’-GTGGCGCACTTGCAACTGGC-3’ | 5’-AGAGCGGAATCTGGGTGTCCCA-3’ | |
| YCAE1GL001938 | 3-oxoadipate CoA-transferase | | *oct* | 5’-ATGACCATCACCAAAAAGCTCTCCC-3’ | 5’-CCTTGTCGCCCAGGTAGTTGGC-3’ | |
| YCAE1GL004618 | 4-hydroxyacetophenone hydrolase | | *hah* | 5’-ATGCACGACGTATTCATCTGTGACG-3’ | 5’-CGCCAGGGTTGCGCTCG-3’ | |
| YCAE1GL001881 | Hydroxyquinol 1,2-dioxygenase | | *hyd* | 5’-ATGAGAAATCTGGACGAACACACTA-3’ | 5’-TTGTCTCACGAGCGAAGGC-3’ | |

**Table S2.** Filtered reads quality statistics

| **Sample** | **Total Raw Reads (Mb)** | **Total Clean Reads (Mb)** | **Total Clean Bases (Gb)** | **Clean Reads Q20 (%)** | **Clean Reads Q30 (%)** | **N Reads (%)** | **Low Quality Reads**  **(%)** | **Adapter Reads (%)** | **Other Reads (%)** | **GC content (%)** | **Clean Reads Ratio (%)** |
| --- | --- | --- | --- | --- | --- | --- | --- | --- | --- | --- | --- |
| CK1 | 17.97 | 16.10 | 2.42 | 98.81 | 95.22 | 0.00 | 0.83 | 9.55 | 0.00 | 60.31 | 89.62 |
| CK2 | 16.34 | 14.89 | 2.23 | 98.90 | 95.48 | 0.00 | 1.15 | 7.71 | 0.00 | 60.17 | 91.14 |
| CK3 | 17.97 | 16.07 | 2.41 | 98.91 | 95.50 | 0.00 | 0.94 | 9.61 | 0.00 | 60.16 | 89.45 |
| T1 | 17.97 | 15.90 | 2.39 | 98.75 | 95.07 | 0.00 | 1.09 | 10.40 | 0.00 | 60.15 | 88.51 |
| T2 | 17.97 | 15.82 | 2.37 | 98.84 | 95.31 | 0.00 | 1.01 | 10.95 | 0.00 | 59.87 | 88.05 |
| T3 | 16.34 | 14.74 | 2.21 | 98.77 | 95.14 | 0.00 | 0.97 | 8.80 | 0.00 | 59.59 | 90.23 |

**Table S3.** Statistics of New Transcript Types

| **Class** | **Coding** | **Non-Coding** | **Total** |
| --- | --- | --- | --- |
| All | 107 | 1,298 | 1,405 |
| Intergenic region (IGR) | 63 | 710 | 773 |
| Antisense to mRNA (AM) | 44 | 588 | 632 |

| **Table S4.** Summary of genes encoding enzymes involved in conversation of bisphenol A degradation products | | | | | |
| --- | --- | --- | --- | --- | --- |
| **GeneID** | **EC number** | Enzyme recommended name | Abbrevation | Substrate | Product |
| YCAE1GL000629 | **1.2.1.18 1.2.1.27** | 4-hydroxybenzaldehyde dehydrogenase | hbd | 4-hydroxybenzaldehyde | 4-hydroxybenzoate |
| YCAE1GL003062 | **1.14.13.2** | 4-hydroxybenzoate 3-monooxygenase | hbm | 4-hydroxybenzoate | 3,4-dihydroxybenzoate |
| YCAE1GL004616 | **5.5.1.2** | γ-carboxymucono-lactone hydrolase | clh | β-carboxy-muconatea | γ-carboxymucono-lactone |
| YCAE1GL004614 | **4.1.1.44** | 4-carboxymuconolactone decarboxylase | cmd | γ-carboxymucono-lactone | 3-oxoadipate enol-lactone |
| YCAE1GL004615 | **3.1.1.24** | 3-oxoadipate enol-lactonase | oel | 3-oxoadipate enol-lactone | 3-oxoadipate |
| YCAE1GL004792 | **1.13.11.3** | protocatechuate 3,4-dioxygenase | pcd | 3,4-protocatechuate | β-carboxy-muconatea |
| YCAE1GL001938 | **2.8.3.6** | 3-oxoadipate CoA-transferase | oct | 3-oxoadipate | 3-oxoadipyl-CoA |
| YCAE1GL004618 | **2.3.1.174** | 3-oxoadipyl-CoA thiolase | odt | 3-oxoadipyl-CoA | succinyl-CoA |
| YCAE1GL002120 | **1.14.13.82** | 4-hydroxyacetophenone monooxygenase | ham | 4-hydroxyacetophenone | 4-hydroxyphenyl acetate |
| YCAE1GL004615 | **3.1.1.24** | 4-hydroxyacetophenone hydrolase | hah | 4-hydroxyphenyl acetate | Hydroquinone |
| YCAE1GL001881 | **1.13.11.37** | Hydroquinone dioxygenase | hqd | Hydroquinone | 4-hydroxymuconic semialdehyde |
| YCAE1GL001881 | **1.13.11.37** | Hydroxyquinol 1,2-dioxygenase | hyd | Hydroquinone | Maleylacetate |
| YCAE1GL005307 | **1.2.1.68** | 4-hydroxymuconic semialdehyde dehydrogenase | hmd | 4-hydroxymuconic semialdehyde | Maleylacetate |
| YCAE1GL001916 | **1.3.1.33** | Maleylacetate reductase | mlr | Maleylacetate | 3-oxoadipate |
| YCAE1GL005919 | **1.14.-.-** | Cytochrome p450 monoxygenase | cyp | Bishenol A | 1,2-bis(4-hydroxyphenyl)-2-propanol or 4,4-dihydroxy-alpha-methylstilbene |


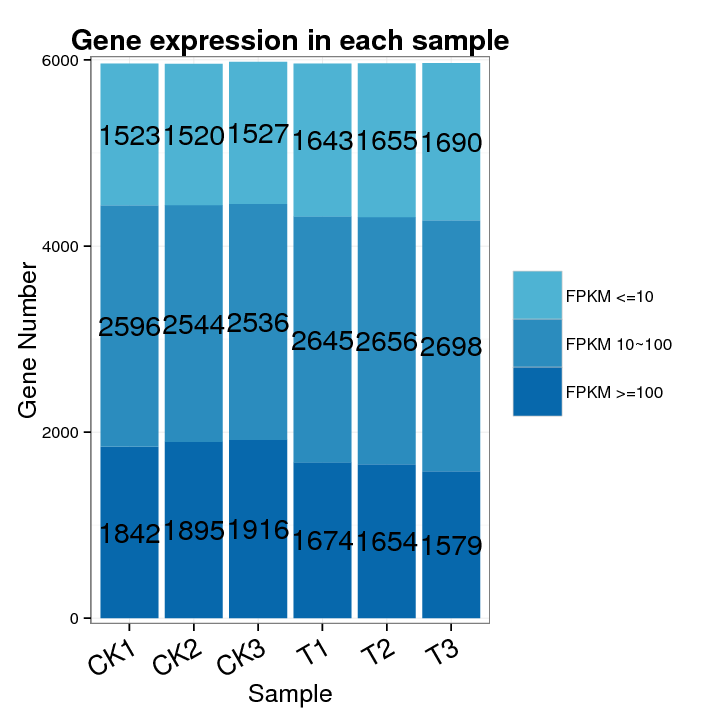


**Fig. S1.** The distribution of gene expression in control (CK) and treated (T) samples shows the number of expressed genes in each sample with a corresponding FPKM value.


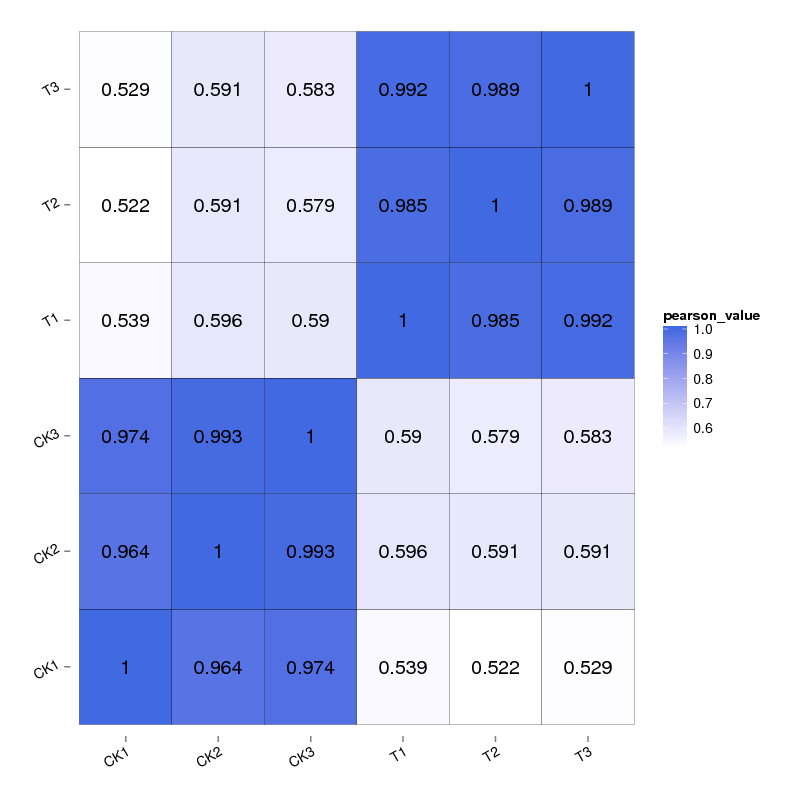


**Fig. S2.** Heat map of correlation between samples


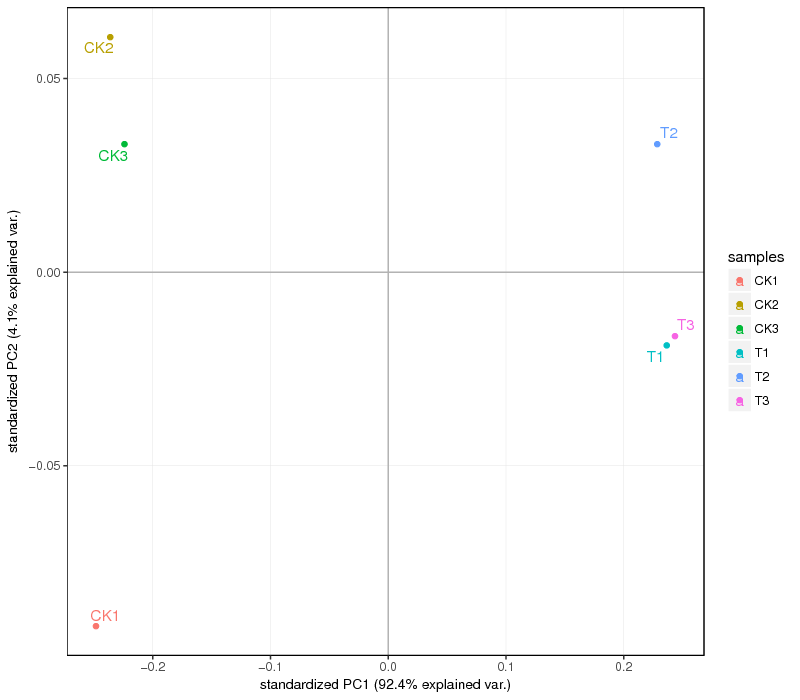


**Fig. S3.** PCA analysis results for control and treated samples.


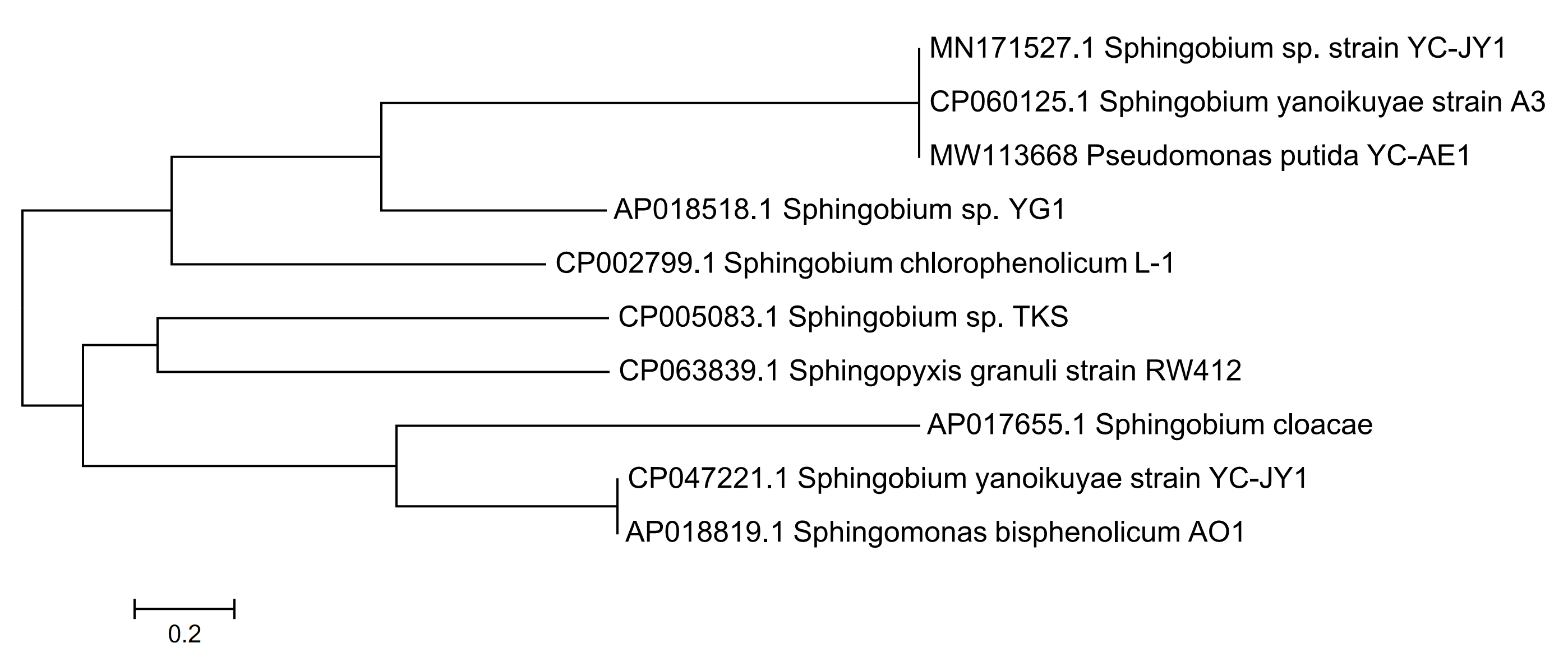


**Fig. S4.** Phylogenetic tree for BisdA gene of *Pseudomonase putida* strain YC-AE1 and other related BisdA genes from other bacterial strains

**RNA extraction method**

1. Take the no more than 109 cells (if the cells were block ground into powder with liquid nitrogen; for positive cells, use 100 μL TE buffer (3 mg/mL) containing lysozyme to completely resuspend the cells and incubate for 5min at room temperature).
2. Add 1.5mL TRIzol, shake vigorously for 3 minutes, and leave it at room temperature for 5 minutes.
3. Centrifuge at 10000rpm for 5min at 4℃.
4. Transfer supernatant into a new 2.0mL EP tube, add 200μL of chloroform/isoamyl alcohol per mL of lysate, and mix by inverting vigorously.
5. Centrifuge at 10000rpm at 4℃ for 10min.
6. Pipette the supernatant into a new 1.5mL centrifuge tube. Be careful not to absorb the middle protein layer. Add isopropanol equal to the volume of the supernatant, and gently invert to mix.
7. Precipitate in the refrigerator at -20°C for 1 hour.
8. Centrifuge at 13600rpm for 20min at 4°C.
9. Collect the supernatant, add 1mL of 75% ethanol, purge and settle with a pipette.
10. Centrifuge at 10000 rpm and 4°C for 3 minutes, discard the supernatant, centrifuge briefly, discard the remaining liquid, and dry for 3-5 minutes.
11. Dissolve the precipitate with 30-100μL DEPC water or RNase-free water.

**Protein sequences**

>BisdA sequence (gene bank protein ID: QRZ20881.1)

MPHIQVTTRDGEIRELDVAASGFLMEALRDANIDGVEAICGGCCSCATCHVYIDAAPAGTLPPVSSDEEMLLSGLVSTPGRSRLSCQIPVTAELDGLKLTIPPDS.

>BisdB sequence (gene bank protein ID: QRZ20882.1)

MNPQTLPVFPDLDIFSPEYACNREKYAARALRDYPLHFYKPLNLWIVSKHKDVRSALFTPQVFSSVAFGLLPPPDDIAPRVPDLYTDVHLPSMDPPEHTKLRVPVQQALLPGRLVGKDEVVRRIANELIDTFIDKGECDLLHDFSYKLALYLIVDMLGLPKERAEDYHRWSNCFFQLFTPKVPERADARFFVPMPEEVLRQNWEDLAEANDYLREVVENLDRNPGNNMLSNLLQLREPDGSRTITISANVRNALEFGAAGHDTTATLIAHLTYFVLTTPDLKDTLTEDPSLIPAAISETLRRRGSVDGLFRRTLSDVELCGQKIESGSIVYLDLTAANLDPDVFPEPETFRLNRDNIKEMVSFGYGRHVCAGQYLSRIEAKAAYEELMRRIPNMRLADGFKLEYMPSVATTVLKGLPLVWDKN.
